# Using real patients in e-learning: case-based online training in child and adolescent psychiatry 

**DOI:** 10.3205/zma001389

**Published:** 2020-12-03

**Authors:** Regina Taurines, Franziska Radtke, Marcel Romanos, Sarah König

**Affiliations:** 1 University Hospital Würzburg, Center of Mental Health, Department of Child and Adolescent Psychiatry, Psychosomatics and Psychotherapy, Würzburg, Germany; 2 University Hospital Würzburg, Institute of Medical Teaching and Medical Education Research, Würzburg, Germany

**Keywords:** case-based online training, e-learning, child and adolescent psychiatry, pandemic

## Abstract

**Objectives: **In undergraduate medical education and in the subject of child and adolescent psychiatry, examining young patients face-to-face is a key element of teaching. With the abrupt shutdown of face-to-face teaching caused by the SARS-CoV-2 pandemic, a case-based online training program integrating audio and video of real patients was developed.

**Methods: **The blended learning platform CaseTrain guides medical students in their final year through real child-psychiatric patient cases, such as anorexia, autism, or attention deficit disorder, through presentation of video and audio of real patients and parents. The teaching format complements lectures on child psychiatric topics, comprising asynchronous elements (self-study using the digital material) as well as synchronous elements (web-conferences with a specialist). Learning objectives for students were set to develop knowledge of the spectra of psychiatric disorders that affect children and to recognize approaches how to assess and manage common psychiatric problems of childhood and adolescence.

**Results: **The feedback from medical students through oral and written evaluation was positive. They appreciated getting to know ‘real-world patients’ in times of such a pandemic, to learn explorative techniques from role models, and to be in close contact with the supervising specialist. In consequence of critical feedback on the length of some video sequences, these training units will undergo revision.

**Conclusions: **Case-based online training may continue to be a useful option in a post-pandemic future as integral part of medical education, complementing face-to-face lectures and training in (child) psychiatry.

## Zusammenfassung

**Zielsetzungen:** Im Studium der Humanmedizin und besonders im Fach Kinder- und Jugendpsychiatrie ist die Untersuchung junger Patienten von Angesicht zu Angesicht ein Schlüsselelement der Lehre. Mit der abrupten Einstellung der Präsenzlehre aufgrund der SARS-CoV-2-Pandemie wurde ein fallbasiertes Online-Trainingsprogramm entwickelt, in das Audio- und Videoaufnahmen von realen Patienten integriert wurden.

**Methoden: **Auf der Plattform CaseTrain, eingebettet in ein Blended-Learning-Konzept, bearbeiten Medizinstudierende kinderpsychiatrische Patientenfälle, beispielsweise zu den Themen Anorexie, Autismus oder Aktivitäts- und Aufmerksamkeitsstörung. Videos und Audioaufnahmen realer Patientinnen und Patienten und deren Eltern sind in die Fälle integriert. Das Online-Lehrangebot für Studierende im letzten Studienjahr vor dem Praktischen Jahr wird ergänzt durch Vorlesungen zu kinderpsychiatrischen Themen und enthält sowohl asynchrone (Selbststudium unter Verwendung des digitalen Materials) als auch synchrone Elemente (Web-Konferenzen mit einer fachärztlichen Person oder erfahrenen Psychologinnen und Psychologen). Die Lerninhalte umfassen das gesamte Spektrum psychischer Störungen im Kindes- und Jugendalter. Ferner werden Kompetenzen zu häufigen psychischen Problemen im Kindes- und Jugendalter im Hinblick auf die klinische Einordnung und zielgerichteter diagnostischer und therapeutischer Verfahren vermittelt.

**Ergebnisse: **Das Feedback durch die Medizinstudierenden mittels mündlicher und schriftlicher Evaluationen war positiv. Sie schätzten es, in Pandemie-Zeiten den "Patienten aus der realen Welt" virtuell zu begegnen, Gesprächsmethoden am Vorbild zu erlernen und sich mit den Lehrpersonen auszutauschen. Infolge der kritischen Rückmeldung zur Länge einiger Videosequenzen werden diese Ausbildungseinheiten überarbeitet.

**Schlussfolgerungen: **Das hier vorgestellte fallbasierte Online-Trainingsprogramm kann auch in Zukunft nach der Pandemie ein wertvolles Lehrangebot darstellen und Präsenzveranstaltungen in der (Kinder- und Jugend-)Psychiatrie – wie Vorlesungen und Praktika – im Sinne eines integrierten Ausbildungsansatzes ergänzen.

## 1. Objectives

Real-patient cases are known to be effective in developing students' self-directed learning skills, improving their confidence in future patient encounters, and fostering knowledge gains [1]. In undergraduate education and in the subject of child and adolescent psychiatry in particular, assessing the history of young patients face-to-face, observing their mood, social interaction, and behavioral characteristics are key elements of teaching. Thus, lectures and seminars usually include live interaction between the examiner and patients providing authenticity and enhanced instructiveness considered as crucial advantages by students [2]. With the abrupt shutdown of face-to-face teaching in the course of the SARS-CoV-2 pandemic, we developed a case-based online training program, integrating audio and video of real patients aiming to transfer the real-life experience into an online format. The case-based learning approach was chosen and transferred online to ensure the exposition of students to real-world patient scenarios despite the given absence in clinic [3]. The aim of the online training was to enhance students’ existing theoretical knowledge and to develop their clinical reasoning skills in common psychiatric disorders that affect children [4].

## 2. Method: case-based online training

Within CaseTrain, a blended learning platform established at the University of Würzburg [5], [6], [7], medical students are presented with real child-psychiatric patient cases presenting different mental diseases (see table 1 (Tab. 1)).

**Figure 1:**
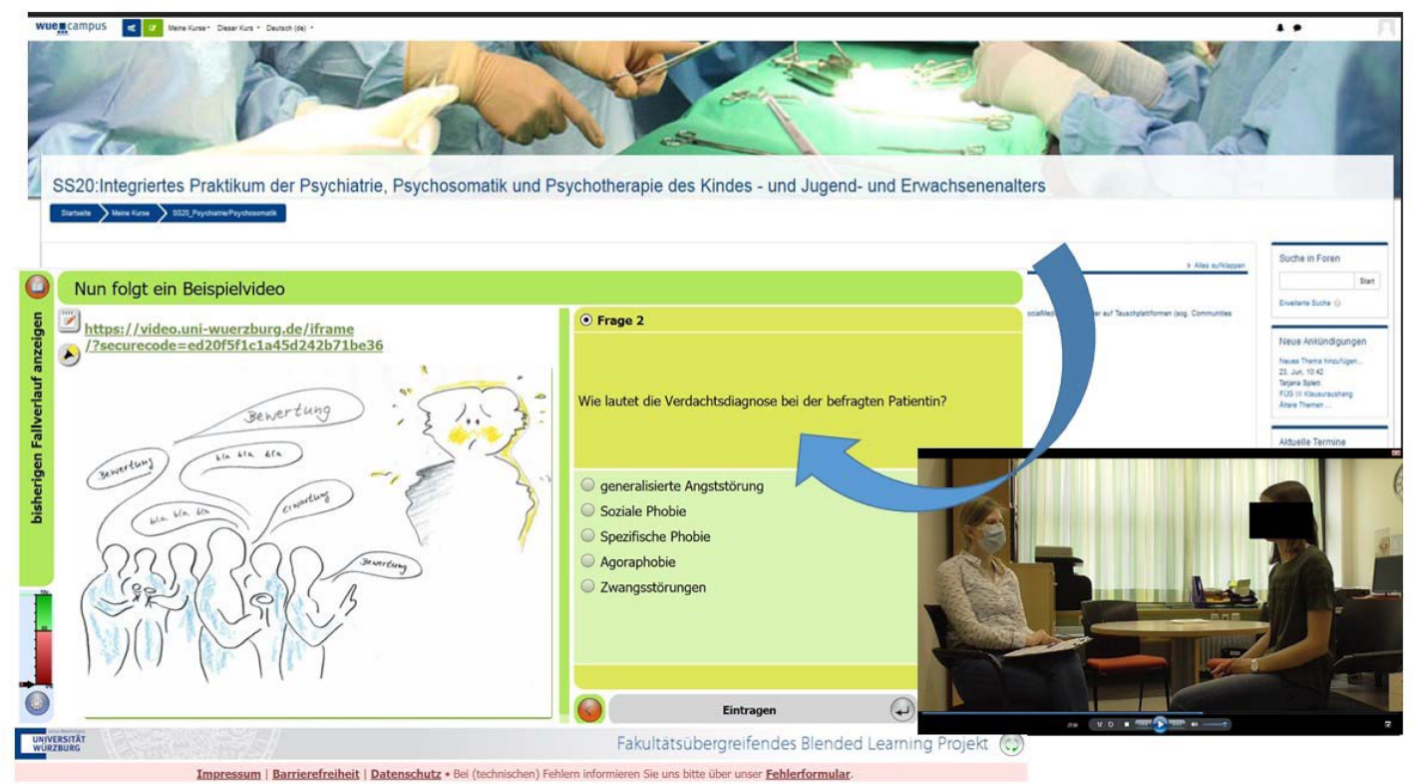
CaseTrain “anxiety disorders”, sequence on ‘social phobia’ including a patient presentation during the SARS-CoV-2 pandemic.

**Figure 2:**
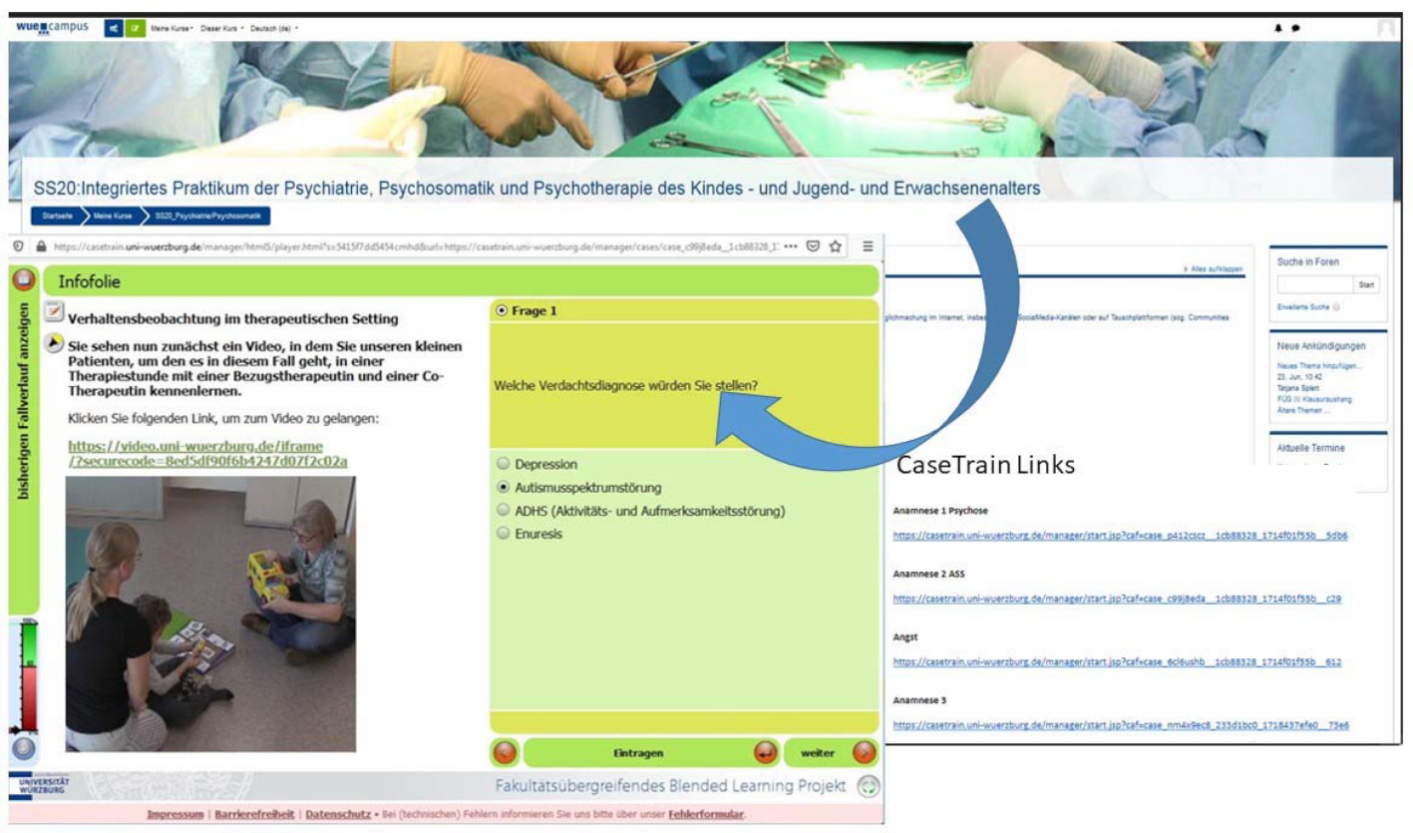
CaseTrain video “autism spectrum disorder”, sequence of an autism-specific therapy session with two therapists using picture cards for communication.

We produced video and audio sequences while interviewing patients and parents/carers – complemented in a few cases with simulated patients from the medical team. These sequences included interviews on the medical history (see figure 1 (Fig. 1)) and explorations into psychopathological aspects, each with a duration of 10 to 40 minutes. Other videos presented characteristic behavioral features and treatment methods during a therapy lesson, e.g. with an autistic child (see figure 2 (Fig. 2)). All EU data protection laws (GDPR) were applied.

The online training replaced a compulsory course for students in their fifth year of the six-year degree course in human medicine. The training concept comprised an asynchronous and a synchronous element.

1. **Asynchronous: **Students were provided digital material via the local learning platform WueCampus including audio and video as well as excerpts of the patients’ charts such as their history and the clinical presentation of the disorder. Students were instructed to perform literature research on the topic. As self-assessment, the CaseTrain units included multiple-choice and open questions with automated feedback.
2. **Synchronous: **Students attended web conferences of 45 minutes’ duration on each case in small groups with a specialist or consultant in child psychiatry/child psychology. They discussed their results from self-study, and reflected on their learning experiences. The supervising specialist revised the patient case, gave information on the patient’s further development, as well as details on psycho-/pharmacotherapy.

During the web conferences, the underlying educational model was to compile the students’ hypothesis generation, pattern recognition, context formulation, diagnostic method interpretation, differential diagnosis, and diagnostic verification approaches as key elements of clinical problem solving [8]. The detailed learning objectives of the online training (parts A and B) are listed in table 1 (Tab. 1).

Students were asked for feedback at the end of each web conference. Additionally, they rated their satisfaction with the online training by means of an online evaluative survey (5-point Likert scale: 1=strongly agree, 5=strongly disagree). 

## 3. Results

We experienced a great commitment of patients and families as well as the staff in their respective contributions to the design of the CaseTrain units. Student feedback has been highly positive, pointing out the experience of real patients in times of the pandemic, the deeper insight into child psychiatry, the explorative techniques from role models, and the close contact with the teaching consultant. Based on the verbal feedback on the length of some video sequences, training units are being revised where appropriate.

A total of 23 students (21.3% of the total semester) participated in the survey. They rated the item “The materials and media were suitable to achieve the goals set” with a mean value of 1.4±0.6 and the item “On the basis of my previous knowledge, I have learned a lot” as 1.4±0.8. The highest item rating of 1.1±0.5 was achieved for “I had the opportunity to exchange with the teaching staff and ask questions”. This online training program was ranked second place in the faculty ranking of all the clinical courses taught within the degree program.

The supervising specialists experienced the success of online meetings to be dependent on the active participation of students. Hence, reducing group sizes and including role play may prove beneficial. Technical problems were scarce, since the contact details for technical support were available in advance. 

## 4. Conclusions

During the SARS-CoV-2 pandemic, students were grateful for the opportunity to study with real cases that the program provides, as they reflect polymorphisms of actual clinical material encountered in everyday practice [8]. The online training program presented here may remain as a useful tool in a post-pandemic future, complementing face-to-face lectures and practical training sessions. 

Online cases may enhance a patients’ willingness to participate in teaching, as their presentation can be avoided in large lectures. Furthermore, different phases of the clinical picture and therapeutic progress may be presented, thus promoting knowledge gain. 

We are continuing with the implementation of case-based online training, expanding with additional disorders, and extending the application to courses in preclinical medical school. Further research may also be needed to address the question as to what extent perceived student prejudice with respect to psychiatric illnesses in children and adolescents may be reduced through online courses to a degree replacing the face-to-face contact.

## Competing interests

The authors declare that they have no competing interests. 

## Figures and Tables

**Table 1:**
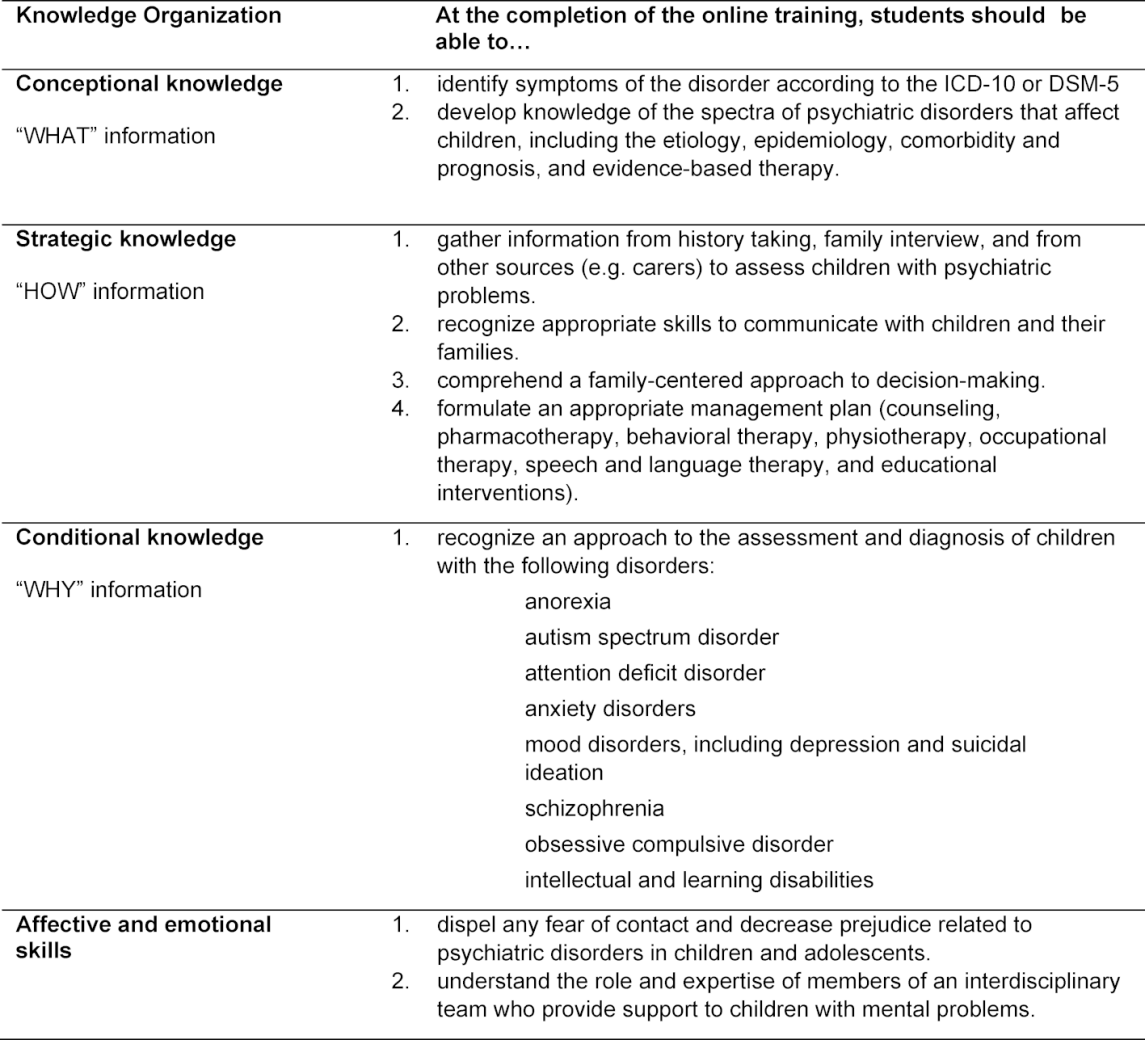
Learning objectives for the child and adolescent psychiatry online training, classified according to the knowledge organization concept of Schmidmaier et al. 2013 [9]
